# Smart Osteology: An AI-Powered Two-Stage System for Multi-Species Long Bone Detection and Classification Using YOLOv5 and CNN Architectures for Veterinary Anatomy Education and Forensic Applications

**DOI:** 10.3390/vetsci12080765

**Published:** 2025-08-16

**Authors:** İmdat Orhan

**Affiliations:** Department of Anatomy, Faculty of Veterinary Medicine, Erciyes University, 38039 Kayseri, Türkiye; imdatorhan@erciyes.edu.tr

**Keywords:** veterinary anatomy, anatomy education, bone classification, artificial intelligence, deep learning

## Abstract

In this study, bone detection was performed using the YOLO algorithm on a dataset comprising photographs of the scapula, humerus, and femur from cattle, horses, and dogs. Subsequently, convolutional neural networks (CNNs) were employed to classify both the bone type and the species. Trained on a total of 26,148 images, the model achieved an accuracy rate of up to 97.6%. The system was designed to operate not only on mobile devices but also in an offline, “closed model” version, thereby enhancing its applicability in forensic medicine settings where data security is critical. Additionally, the application was structured as a virtual assistant capable of responding to users in both written and spoken formats and of generating output in PDF format. In this regard, this study presents a significant example of digital transformation in fields such as veterinary anatomy education, forensic medicine, archaeology, and crime scene investigation, providing a solid foundation for future applications.

## Simple Summary

Understanding which animal a bone belongs to is important in many areas, such as veterinary education, archaeology, and forensic science. In this study, I developed a smart system that uses artificial intelligence to identify both the type of bone and the animal species it comes from. I used thousands of bone images from cows, horses, and dogs to train the system. The system can work on mobile phones and does not need an internet connection, which makes it useful in the field. Students tested the system and found it easy to use and helpful for learning. This technology can make anatomy education more accessible and help experts in different fields quickly identify bones. It is a practical and innovative tool that brings the power of artificial intelligence to everyday educational and professional use.

## 1. Introduction

Artificial intelligence (AI), particularly deep learning techniques, has led to significant advancements in numerous fields, including veterinary medicine, human medicine, and forensic sciences in recent years. Deep learning architectures such as convolutional neural networks (CNNs) have been successfully applied in medical imaging with high accuracy, contributing to fracture detection, organ segmentation, and tumor classification [1,2]. In veterinary medicine, AI-based systems have begun to be used for the analysis of radiographic images, enabling fracture diagnosis, abnormality detection, and early identification of certain internal diseases [3,4].

Recent developments in deep learning have led to significant improvements in bone detection and classification tasks, particularly through the use of YOLO (You Only Look Once)-based object detection algorithms. Tariq and Choi [5] demonstrated that an enhanced YOLO11 architecture could detect and localize wrist fractures in X-ray images with high precision, underscoring the diagnostic power of real-time convolutional networks in skeletal image analysis.

Research on deep learning has enabled efficient segmentation of skeletal structures in veterinary imaging. For instance, Kvam et al. [6] demonstrated the feasibility of using convolutional neural networks (CNNs) to automate the identification and segmentation of pig skeletons from CT scans, highlighting the growing potential of AI in animal anatomy research.

Recent advances in deep learning have enabled accurate assessments of skeletal development and trauma timing, even in non-human species. For example, Ergün and Güney [7] demonstrated the use of convolutional neural networks for classifying bone maturity and fracture age in canine long bones using radiographic images.

Multi-view deep learning models have shown promise in improving the accuracy of radiographic interpretation in veterinary medicine in recent years. For instance, Dourson et al. [8] developed the VET-DINO system, which demonstrated enhanced classification performance by leveraging multi-angle images in the analysis of veterinary radiographs.

However, the use of AI in the classification and automated identification of animal bones remains limited. Most existing studies have focused on human medicine, often addressing deformation analysis of human bones [9].

In veterinary anatomical education, osteology holds critical importance for enabling students to learn bone structures and species-specific anatomical features. However, limitations in hands-on laboratory instruction and dependence on instructor availability may hinder the learning process. It has been reported that receiving real-time feedback can improve retention in learning by up to 40% [10]. AI-supported mobile applications address this need by promoting individualized learning and facilitating digital transformation in educational environments [11,12].

The proliferation of mobile applications in anatomy education has opened new avenues for student engagement; however, the pedagogical rigor and scientific credibility of these tools vary widely. Rivera García et al. [13] emphasized that while many anatomy apps are popular, few are developed within academic contexts or validated through structured evaluation methods.

The growing use of mobile applications in human anatomy education has prompted critical evaluations of their pedagogical effectiveness. Rivera García et al. [13] emphasize that while such apps enhance accessibility, their anatomical accuracy and scientific validation often remain questionable.

Recent large-scale studies have shown that screen-based 3D and augmented reality tools can significantly improve student engagement and learning experience in anatomy education [14]. These tools provide spatial understanding and interactivity, which are especially useful in visual-heavy disciplines like anatomy.

Latest developments in computer vision have led to the development of cloud-based web applications such as ShinyAnimalCV, which facilitate object detection, segmentation, and 3D visualization of animal data [15]. This tool integrates pre-trained vision models and user-friendly interfaces to democratize access to image analysis methods for educators and students alike.

Interactive and augmented reality (AR) tools have emerged as powerful resources in veterinary anatomy education, offering immersive and intuitive experiences that surpass traditional teaching methods. Christ et al. [16] demonstrated this potential by developing a mobile AR application for canine head anatomy, highlighting the feasibility of extending such digital workflows into veterinary curricula.

Emerging technologies such as augmented reality (AR) have been shown to enrich anatomical education by offering interactive and spatially intuitive experiences. Jiang et al. [17] developed an AR-based canine skull model that effectively supported veterinary students’ learning without compromising comprehension when compared to traditional methods.

Conversational AI tools such as ChatGPT have opened new horizons for interactive learning in veterinary anatomy, providing instant explanatory feedback to students [18]. These chatbots have been shown to enhance anatomical knowledge retention while highlighting the continuing importance of hands-on dissection practices.

In disciplines such as forensic science, archaeology, and crime scene investigation, the rapid and accurate determination of whether a bone belongs to a human or an animal is of paramount importance. However, the absence of experts in field situations can delay the process and increase the likelihood of errors [19,20]. In such cases, AI-based systems may serve as supportive tools that augment expert decision-making without replacing human specialists.

The aim of this study was to train an artificial intelligence system using image processing methods to recognize the scapula, humerus, and femur of cattle, horses, and dogs, and to evaluate the system’s performance in identifying these bones through a custom-developed application.

## 2. Materials and Methods

### 2.1. Study Design and Data Collection

In this study, scapula, humerus, and femur belonging to cattle (*Bos taurus*), horses (*Equus caballus*), and dogs (*Canis familiaris*) were utilized. The bone images were obtained from specimens available in the Anatomy Laboratory of the Faculty of Veterinary Medicine at Erciyes University.

Two stages were defined during the preparation of the dataset. In the first stage, the YOLO model was trained to distinguish bone structures from other objects using a limited number of images containing bones from various species, including those not present in our main dataset (Table 1). As a result, there was no need for additional cropping when creating the classification dataset.

**Figure 1:**
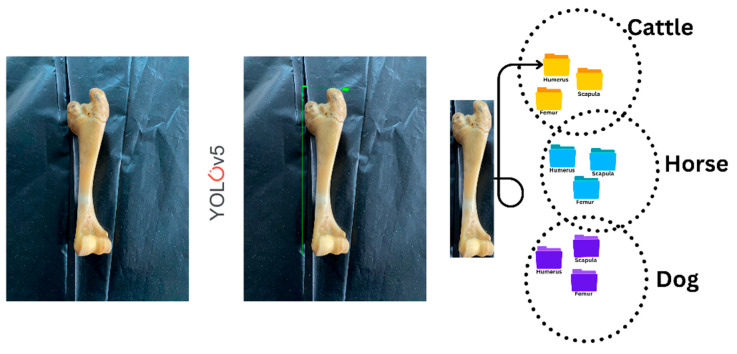
Working process of the YOLO system.

The trained YOLO model was used to scan bone images placed sequentially on a table from various angles, and the cropped images were recorded under the corresponding animal class (Figure 1).

A total of 26,148 bone images were collected. Of these, 24,700 images were used for training and testing the model, while the remaining images were reserved for external validation. A systematic data collection protocol was implemented to ensure balanced representation across all classes. Regardless of the number of available physical bone specimens, exactly 2744 images were acquired for each bone–species combination (Table 2). For categories with fewer bone samples (e.g., dog humerus: 38 bones), multiple images were captured from different angles, lighting conditions, and positions to reach the target number. For categories with more bone samples (e.g., cattle scapula: 62 bones), fewer images per bone were taken while maintaining diversity. This approach ensured equal representation across all nine classes and prevented model bias toward categories with a higher number of physical specimens.

All images were captured at 720p resolution and were subsequently cleaned to remove outliers. Care was taken to balance the dataset according to bone type and species. To enhance model performance and generalizability, data augmentation techniques such as brightness adjustment, rotation, cropping, and horizontal flipping were applied.

### 2.2. Image Processing and Annotation

**Figure 2:**
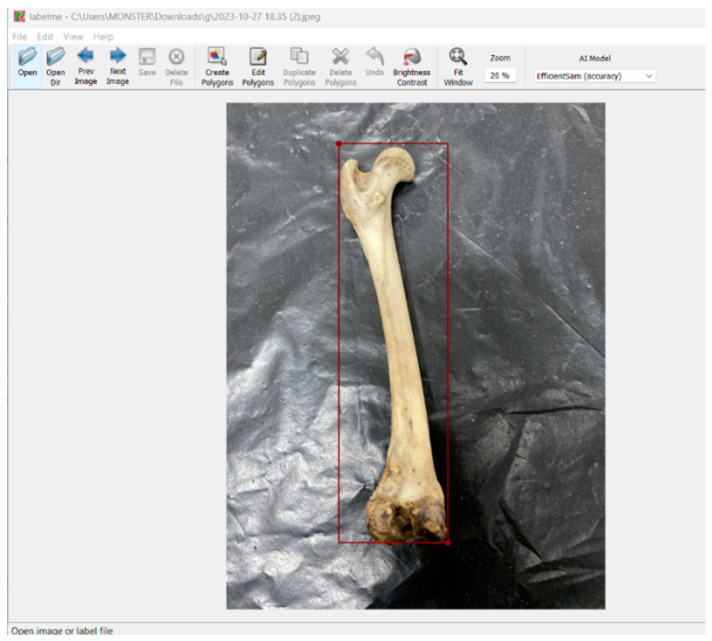
Screenshot of the bone annotation process (using Labelme).

The bone images were initially processed using the YOLOv5 algorithm, which automatically detected the relevant regions of interest. For the annotation process, the Labelme platform was utilized (Figure 2), and distinct anatomical features of each bone (e.g., *processus hamatus*) were manually marked. The annotated data were exported in COCO JSON (Common Objects in Context) format for further use.

### 2.3. Deep Learning Architecture and Model Training

The dataset was divided into three subsets: training (85%), testing (10%), and validation (5%). Model training was conducted using the Python (version 3.12.0) programming language and the PyTorch (version 2.8.0) framework. In the initial stage, bone detection was performed using the YOLO algorithm. Subsequently, bone name and species classification were carried out using CNN architectures. The training process lasted approximately seven hours in total.

#### 2.3.1. Model Selection and Rationale

In this study, a two-stage approach was adopted for bone detection and classification. In the first stage, the YOLOv5 algorithm was employed for object detection. In the second stage, species and bone type classification were performed using the ResNet34, SmallCNN, and AlexNet architectures, which were comparatively evaluated.

##### YOLO Architecture Configuration

YOLO algorithms are known for performing both object localization and classification tasks with high speed and accuracy using a single-stage approach.

In this study, YOLOv5s (small) variant was selected for its optimal balance between detection accuracy and computational efficiency, particularly suitable for mobile deployment requirements. Two main factors influenced the selection of the YOLOv5 model. First, YOLOv5 integrates the Cross-Stage Partial Network (CSPNet) structure into Darknet, establishing CSPDarknet as its backbone architecture [21]. CSPNet addresses the problem of duplicated gradient information encountered in large-scale backbone architectures by incorporating gradient variations in feature maps. This reduces model parameters and floating-point operations per second (FLOPS) while maintaining both inference speed and accuracy, effectively minimizing model size. Speed and accuracy are crucial in the detection of bone structures.

**Figure 3:**
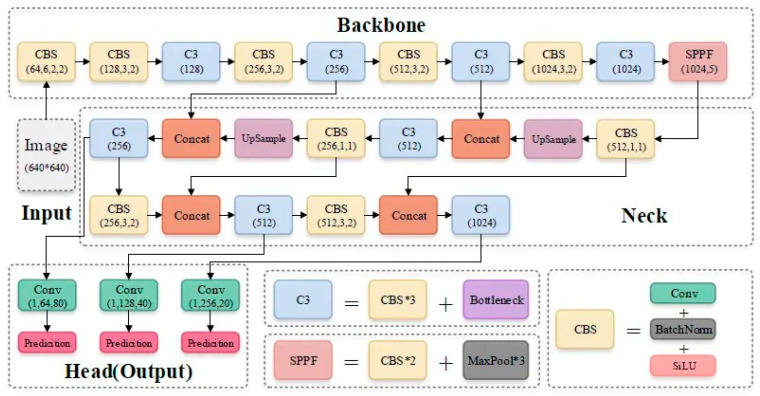
Schematic of the YoloV5 mechanism [23].

Secondly, to enhance information flow, YOLOv5 employs a Path Aggregation Network (PANet) in its neck section [22]. PANet improves low-level feature propagation using a bottom-up approach and introduces a new Feature Pyramid Network (FPN) topology. Additionally, an adaptive feature pooling method connects the feature grid across all feature levels, allowing for meaningful information obtained from each feature level to be transferred to subsequent subnetworks. Furthermore, the YOLO layer, which forms the head of YOLOv5, generates feature maps at three different scales to enable multi-scale prediction, enabling the model to process small, medium, and large-sized objects effectively (Figure 3).

##### CNN Classification Architecture

Resnet34

ResNet34 is a 34-layer deep convolutional neural network architecture developed by Microsoft Research [15]. This architecture utilizes residual connections to address the vanishing gradient problem commonly encountered in deep networks. The key factors influencing the selection of ResNet34 in this study are as follows:

The Residual Learning approach focuses on learning residual functions instead of directly modeling complex functions, as is the case with conventional CNNs. This approach is based on the principle that optimizing identity mappings is easier than optimizing complex residual functions. Through skip connections, information can be transferred directly from lower to higher layers, facilitating better gradient propagation.

**Figure 4:**
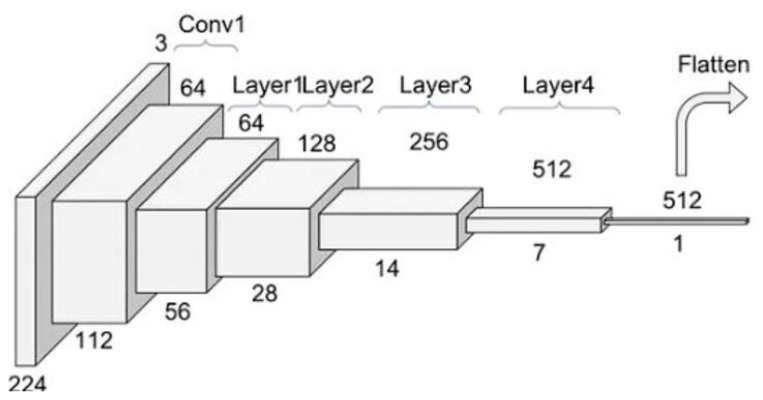
Schematic of the ResNet34 mechanism [24].

The deep structure of ResNet34 makes it a strong candidate for recognizing the complex features of bone morphology. It enables multi-level feature extraction, which is essential for capturing the fine details of anatomical structures (Figure 4).

Small CNN

**Figure 5:**
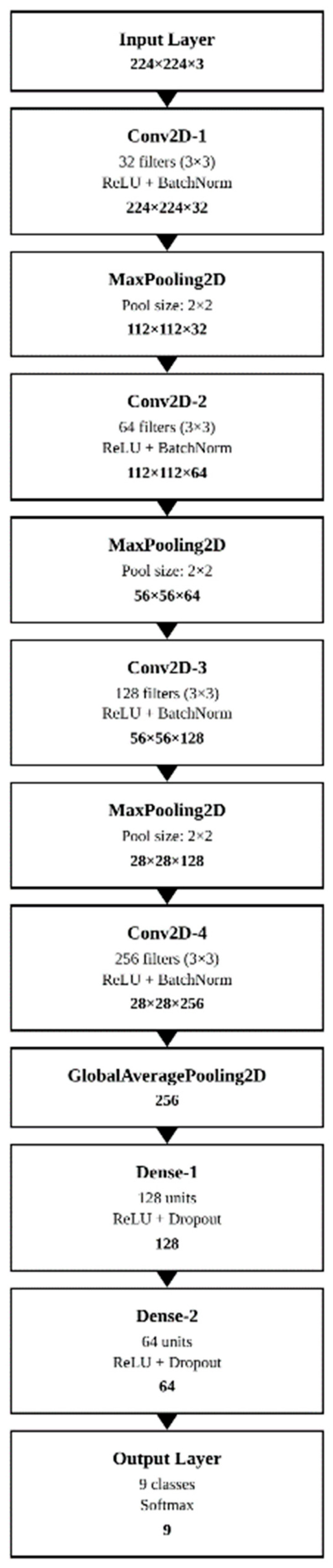
Schematic of the SmallCNN mechanism.

SmallCNN is a lightweight and efficient convolutional neural network architecture specifically designed for this study. This architecture was developed to enable fast inference on mobile devices and to achieve high performance under limited computational resources. SmallCNN is based on the principle of achieving maximum performance with a minimal number of parameters. This approach helps prevent unnecessary complexity, particularly in relatively simple visual recognition tasks such as bone classification, thereby reducing the risk of overfitting and enhancing generalization capability. The developed architecture comprised a total of 387,000 parameters (Figure 5).

AlexNet

**Figure 6:**
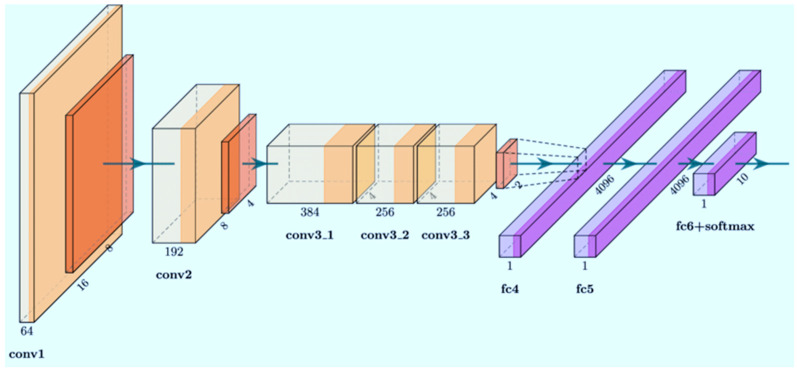
Schematic of the AlexNet mechanism [26].

AlexNet is a convolutional neural network architecture developed by Alex Krizhevsky, Ilya Sutskever, and Geoffrey Hinton in 2012 [25]. This architecture won the first place in the ImageNet Large Scale Visual Recognition Challenge (ILSVRC) 2012 with a top-5 error rate of 15.3%, marking the beginning of the deep learning revolution in the field of computer vision. The primary reason for selecting AlexNet as a comparative architecture in this study is its pioneering role in integrating innovative techniques that have laid the foundation for modern CNN architectures (Figure 6).

The use of the ReLU activation function instead of sigmoid and tanh functions reduced the vanishing gradient problem, enabling faster training. The incorporation of the dropout technique addressed the overfitting problem, particularly enhancing the model’s generalization performance on limited datasets. The AlexNet architecture consists of eight layers: five convolutional layers and three fully connected layers. With approximately 60 million parameters, it provides sufficient learning capacity to capture distinctive features of bone structures without introducing excessive complexity.

#### 2.3.2. Training Configuration and Hyperparameters

During the training of the YOLOv5s model, the input image size was set to 640 × 640 pixels. Although the training was initially scheduled for 150 epochs, an early stopping mechanism was triggered at the 65th epoch due to the stabilization of performance metrics and to prevent the risk of overfitting. The Adam optimizer was employed with an initial learning rate of 0.01 and a momentum value of 0.937. To enhance the accuracy of bone detection, several data augmentation techniques were applied: mosaic augmentation (100%) combined multiple bone samples into a single image to improve the model’s generalization capability; the mix-up technique (15%) mitigated overfitting by smoothing transitions between classes; and manipulations in the HSV color space (H: 0.015, S: 0.7, V: 0.4) improved robustness against varying lighting conditions. All hyperparameters are detailed in Table 3.

Model training was conducted using standardized hyperparameters to ensure a fair comparison across all CNN architectures. All models were trained using the Adam optimizer with a learning rate of 0.001, where the β_1_ and β_2_ parameters were set to 0.9 and 0.999, respectively. To optimize memory usage, the batch size was set to 32, and training was carried out for 100 epochs. Early stopping with a patience value of 15 was applied to prevent overfitting.

To ensure L2 regularization across all models, a weight decay value of 1e-4 was applied. The learning rate was systematically reduced using the StepLR scheduler with a step size of 30 epochs and a gamma factor of 0.1. To address potential class imbalance issues within the dataset, the cross-entropy loss function with balanced class weighting was utilized.

Data augmentation strategies were applied consistently across all models. Brightness adjustment (±20%), random rotation (±15 degrees), and horizontal flipping with a 50% probability were performed. Images were initially resized to 256 × 256 pixels, followed by random cropping to 224 × 224 pixels. ImageNet normalization statistics were applied to the RGB channels, using mean values of [0.485, 0.456, 0.406] and standard deviation values of [0.229, 0.224, 0.225] (Table 4).

The training was conducted on the Google Colab platform using an NVIDIA Tesla T4 GPU (16 GB VRAM), an Intel Xeon CPU, and 12 GB of system RAM. The implementation was developed using the PyTorch 1.12.0 framework with CUDA 11.6 support for cloud-based GPU acceleration.

### 2.4. Model Evaluation

The model’s performance was evaluated not only based on accuracy but also using precision, recall, and F1-score metrics. These metrics provided more reliable insights, particularly in the context of imbalanced datasets. To account for variations in sample size among species, class-weighted F1-scores were calculated. Additionally, the model’s accuracy was assessed using an independent test dataset.

### 2.5. Mobil Application

The trained system was adapted for student use in a laboratory setting. Students access the webpage using the developed link and take a photo of the bone using the “Take Photo” button within the app. They then click the “Analyze” button, and the system analyzes the bone and reports the results below. Students can access the details of the bone within the app if they wish.

### 2.6. Student Surveys

The surveys included informed voluntary consent forms, questions related to the study topic, a brief section providing information about the research, and participants’ feedback on learning outcomes after using the application.

The 45 students who participated in the survey and application were actively taking the anatomy course. It was believed that having these students, who were new to the learning process, try the system, and comment on it would demonstrate its contribution to addressing learning difficulties. Additionally, the application and survey were administered to 105 more students, and the final data were generated accordingly. These remaining 105 students were upper-division students who had passed the anatomy course. This prevented any academic pressure or bias.

An anonymous survey was conducted with 150 students who used our mobile application. The purpose of the survey was to assess the students’ level of interest in such mobile applications and their willingness to use them over an extended period. Additionally, the survey aimed to evaluate the functionality and user-friendliness of the application. The survey included five Likert-scale questions. Students responded using a 5-point Likert-type scale, with 1 indicating “strongly disagree,” 2 indicating “disagree,” 3 indicating “neutral,” 4 indicating “agree,” and 5 indicating “strongly agree.”

### 2.7. Statistical Analysis

The collected data were analyzed using IBM SPSS Statistics software, version 28.0 (IBM Corp., Armonk, NY, USA), and the threshold for statistical significance was set at *p* < 0.05. Mean values and standard deviations (SDs) were reported for satisfaction survey data and demographic characteristics. The normality of data distribution was assessed using the Shapiro–Wilk test. For data that did not show a normal distribution, non-parametric tests were preferred. Satisfaction scores across five different academic levels (from first-year to fifth-year students) were compared using the Kruskal–Wallis H test. The Mann–Whitney U test was applied to compare mean satisfaction scores between gender groups. Effect size calculations were reported using Cohen’s d and eta-squared (η^2^) values. The internal consistency of the survey was evaluated using Cronbach’s α, and the suitability of the factor analytic model was tested using the Kaiser–Meyer–Olkin (KMO) measure and Bartlett’s test.

## 3. Results

### 3.1. Operational Workflow of the Mobile Application

**Figure 7:**
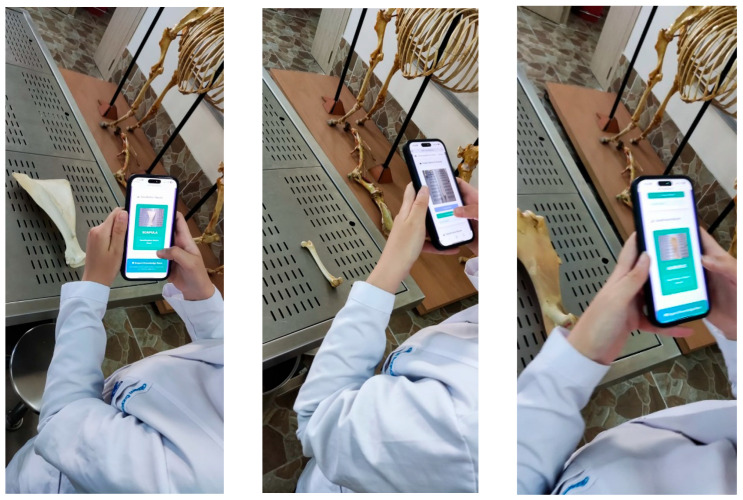
Use of the application by a student in the laboratory.

**Figure 8:**
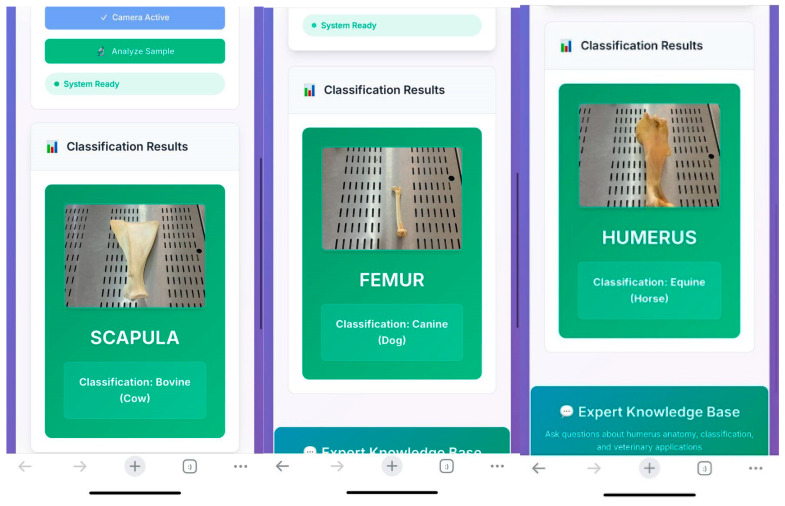
Analysis results generated by the application.

The trained system was used by students in the laboratory setting (Figure 7). Through a web link accessible via mobile phones, students uploaded photographs of bones, which were then rapidly analyzed by the system (Figure 8).

**Figure 9:**
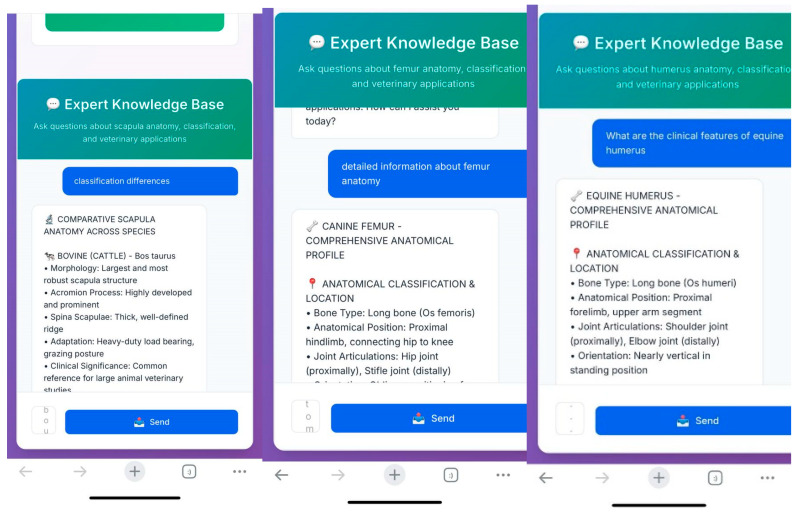
Question-and-answer interface of the system.

Following the analysis, the integrated quick-response system enabled access to additional information regarding the identified bone. Students were able to obtain answers to various questions such as detailed anatomical features of the detected bone, interspecies differences, and the clinical anatomical relevance of the region with pdf format and voice answer (Figure 9).

### 3.2. Yolo Object Detection Performance

As the first stage of our two-step system, the YOLOv5s bone detection model was trained using a dataset comprising 1554 images. The dataset was divided into 85% training (1321 images), 10% testing (155 images), and 5% validation (78 images) subsets, and the training was conducted for 65 epochs. The process was terminated early through an early stopping mechanism once the performance metrics had stabilized.

**Figure 10:**
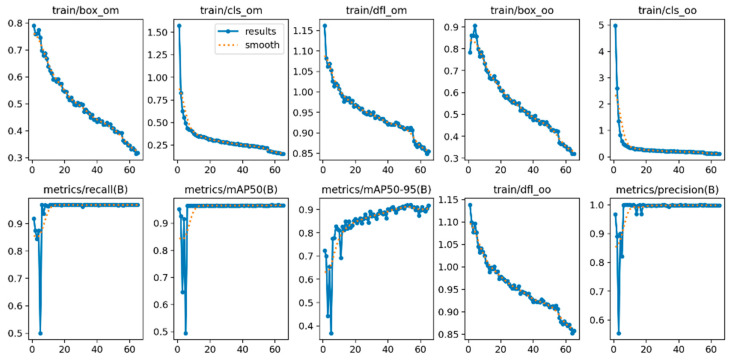
YOLOv5s training metrics over 65 epochs; loss functions (**top row**) and performance metrics (**bottom row**).

Throughout the training process, the loss functions exhibited a consistent decrease, while the performance metrics steadily increased (Figure 10). On the test dataset, the model achieved 96.6% mAP@0.5, 99.8% precision, and 96.9% recall (Table 5).

**Figure 11:**
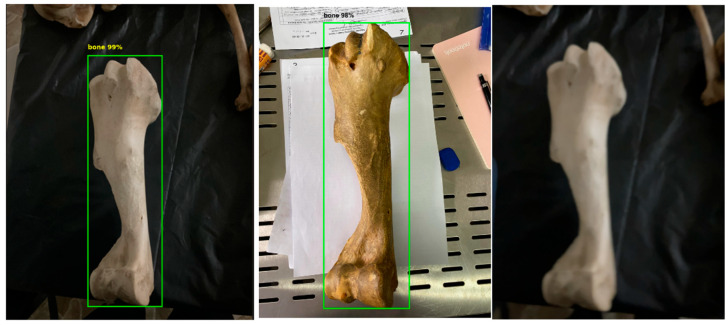
YOLO bone detection results: successful detection with a black background (**left**), detection with a noisy background (**center**), and detection failure due to blurriness (**right**).

The results indicate that the model demonstrated strong generalization capability with minimal overfitting. The consistent performance across the training, testing, and validation sets shows that the YOLOv5s model can reliably distinguish bone structures from the background and provide suitable region proposals for the subsequent CNN classification stage. Furthermore, when the model’s generalization ability was tested, it successfully detected bones even in laboratory images with high background noise (Figure 11). Although this appeared to slightly reduce detection accuracy, it in fact offered an advantage in preparing a high-quality dataset for the CNN classification stage. The automatic filtering of blurred and low-quality images allowed for the subsequent classification model to be trained on cleaner and more reliable data.

### 3.3. Model Performance Comparison

Among the various deep learning architectures trained in this study, the highest classification accuracy was achieved with the ResNet34 model, reaching 97.6%. The alternatively developed SmallCNN architecture achieved an accuracy of 95%. These results indicate that more complex architectures tend to yield higher classification performance (Table 6).

In terms of long bone types, the system demonstrated higher accuracy in recognizing larger bones such as the cow and horse. This suggests that the model was able to distinguish these structures more easily due to their prominent morphological features.

In mobile phone-based applications, the system successfully identified the name of the bones. Notably, it was also able to correctly recognize bones that were not included in the training dataset, demonstrating its ability to generalize to previously unseen samples.

### 3.4. Statistical Analyses of Survey Results

When the responses of the participating students were analyzed according to academic level, no statistically significant differences were found (*p* > 0.05) (Table 7).

**Figure 12:**
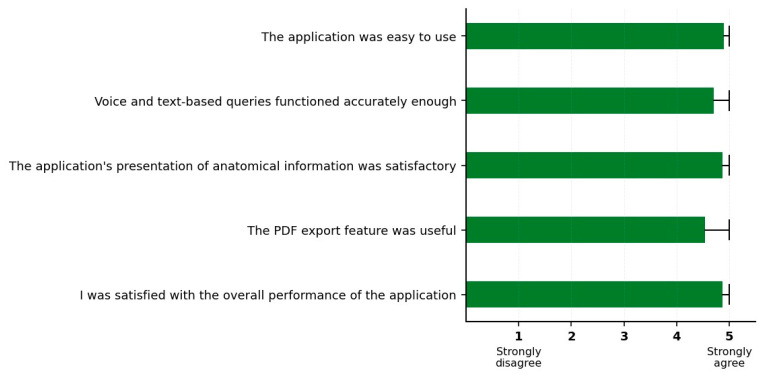
Graphic of the student satisfaction survey (n = 150).

The 5-point Likert scale analysis presented as a horizontal bar chart in Figure 12 and Table 8 comprehensively illustrates the responses of 150 veterinary medicine students regarding the Smart Osteology application. The analysis results show that positive response rates exceeded 90% across all survey items, with the highest satisfaction reported in the “ease of use of the application” category (98.0%).

#### 3.4.1. Descriptive Statistics and Reliability

A total of 150 veterinary medicine students participated in the study, with a mean age of 21.3 ± 2.1 years. Among the participants, 48.7% (n = 73) were male and 51.3% (n = 77) were female. The distribution across academic years was as follows: first-year students 45 (30.0%), second-year students 30 (20.0%), third-year students 25 (16.7%), fourth-year students 25 (16.7%), and fifth-year students 25 (16.7%).

The internal consistency of the survey was assessed using Cronbach’s α, which was calculated as 0.936, indicating a high degree of reliability. The Kaiser–Meyer–Olkin (KMO) measure of sampling adequacy was 0.912, and Bartlett’s test of sphericity was statistically significant (χ^2^ = 1247.82, *p* < 0.001), demonstrating that the dataset was suitable for factor analysis.

#### 3.4.2. Normality Test and Group Comparisons

According to the results of the Shapiro–Wilk normality test, none of the survey items followed a normal distribution (*p* < 0.001); therefore, non-parametric tests were applied. When comparing satisfaction scores across five different academic levels, the Kruskal–Wallis H test revealed no statistically significant differences between the groups (H = 3.892, df = 4, *p* = 0.421, η^2^ = 0.021). This result indicates a small effect size and suggests that academic level does not have a significant impact on application satisfaction.

In the comparison between gender groups, the Mann–Whitney U test showed no statistically significant difference between male students (median = 4.80, IQR = 0.60) and female students (median = 5.00, IQR = 0.40) (U = 2567, *p* = 0.158, r = 0.12). These findings indicate that the Smart Osteology application provides consistent and high satisfaction across both different academic levels and gender groups.

## 4. Discussion

The deep learning-based bone classification system developed in this study demonstrated high accuracy in identifying both bone type and the corresponding animal species. The achieved accuracy rate of 97.6% represents a superior performance compared to previous studies conducted on human bones [2,9]. In particular, the performance attained with the ResNet34 architecture exceeds the commonly reported accuracy range of 90–95% in the literature [19].

The success of the current study in accurately identifying bone types and species aligns with previous research, such as that of Ergün and Güney [7], who highlighted the diagnostic potential of AI in analyzing canine skeletal radiographs. Their work reinforces the notion that automated image-based systems can contribute meaningfully to both veterinary diagnostics and educational settings.

The advantage of incorporating multi-view inputs into AI architectures was also emphasized in the study by Dourson et al. [8], where their model achieved improved accuracy in anatomical interpretation. This aligns with my findings, suggesting that models trained with diverse structural input data can offer more reliable species-level bone classification.

My study aligns with the findings of Kvam et al. [16], as both emphasize the utility of deep learning for precise skeletal identification in non-human species. However, while their approach focused on CT data, our work expands the applicability of AI to photographic images and mobile-friendly platforms, offering broader usability in educational and field settings.

In my study, the implementation of YOLOv5 for detecting bone regions prior to classification builds on this principle, offering reliable and fast identification of anatomical structures from photographs. Similar to the success achieved by Tariq and Choi [5] in clinical radiology, our findings confirm that modern YOLO-based models are highly suitable for veterinary osteological applications where accurate localization is essential.

Existing systems in the literature typically focus solely on human bone analysis and do not address interspecies comparative classification. Although projects such as OsteoID have reported high accuracy, these systems were usually tested on a limited number of species and did not provide publicly accessible datasets [9]. In contrast, the use of a large and diverse dataset comprising bones from different species in the present study enhanced the model’s robustness in real-world applications, where a wide variety of samples may be encountered.

While this study focuses on AI-driven mobile bone classification rather than AR visualization, both approaches share the overarching goal of enhancing student engagement and spatial understanding in anatomy education. The success reported by Christ et al. [16] underscores the value of technology-enhanced learning in veterinary settings, supporting the broader applicability of digital tools like *Smart Osteology*.

While my current system does not yet integrate AR technology, it shares the same goal of enhancing anatomy education through accessible, student-centered digital tools. In line with Jiang et al. [17], our approach also prioritizes learner engagement and real-world usability—especially by providing portable, offline functionality and intelligent anatomical feedback.

While this application does not utilize 3D or augmented reality technologies, it offers interactivity through real-time image analysis, speech-based queries, and dynamic feedback. These features align with the educational benefits highlighted by Barmaki et al. [14], who emphasized that screen-based tools significantly improve student engagement and spatial understanding in anatomy learning.

The 98% satisfaction rate obtained from student surveys further highlights the educational potential of the system. Previous studies, such as those by Mayfield et al. [12], have shown that mobile technologies can effectively support anatomical education. This study not only reinforces those findings but also demonstrates the potential of creating active learning environments that support individualized learning.

Echoing the insights of Choudhary et al. [18], our system embraces AI-assisted feedback by delivering written and spoken anatomical explanations, yet it augments this with image-based bone identification to provide multimodal educational support. By balancing automated instruction with practical application, Smart Osteology addresses both the engagement benefits and limitations of virtual assistants noted by Choudhary et al.

This mobile application responds directly to the concerns raised in Rivera Garcia’s [13] review by providing a scientifically grounded, academically developed system with demonstrable accuracy and user satisfaction. Unlike many commercially produced tools, our app was purpose-built for veterinary anatomical education and forensic support, bridging the gap between innovation and pedagogical reliability as advocated by Rivera García et al. [13].

The analysis results show that positive response rates exceeded 90% across all survey items, with the highest satisfaction reported in the “ease of use of the application” category (98.0%). This finding suggests that students appreciate the use of technology in their educational processes. The relatively lower score for the “PDF export feature” is believed to reflect students’ preference for receiving instant feedback rather than storing or saving information.

When the responses of the participating students were analyzed according to academic level, no statistically significant differences were found (*p* > 0.05). The observation that the mean satisfaction scores of first- and second-year students were higher than those of the other academic levels is thought to be due to the fact that students in the earlier years are still actively engaged in the anatomy education process.

This mobile system extends this concept beyond livestock farming applications by enabling offline bone classification for multiple species directly from device cameras, without the need for internet or cloud infrastructure. While ShinyAnimalCV demonstrates the utility of web-based platforms in academic settings, Smart Osteology prioritizes field-readiness and data privacy through a locally executable, AI-powered app.

## 5. Conclusions

This study successfully demonstrated the applicability of deep learning techniques in classifying certain long bones from selected domestic animal species. Bone detection was performed using the YOLO algorithm, and species and bone name classification were achieved with high accuracy through CNN and ResNet34 architectures. The obtained accuracy rate of 97.6% confirms that the developed system is a reliable tool for both educational and forensic purposes.

As a preliminary study, this work offers a novel and versatile digital solution applicable to fields such as veterinary anatomy education, forensic science, archaeology, and biological anthropology. Future studies aim to expand the scope of the system by increasing the diversity of bone types and animal species, comparing various AI architectures, and integrating interactive technologies such as augmented reality. Additionally, to enhance field applicability, there are plans to further refine user interfaces and establish collaborations with official institutions to develop modules tailored to specific needs.

## Figures and Tables

**Table 1:** Dataset collected for YOLO training.

Animal	Bone	Number of Photos	Right	Left
Horse	Scapula	50	32	18
	Humerus	246	128	118
	Femur	282	92	190
Cow	Scapula	230	122	108
	Humerus	104	40	64
	Femur	172	90	82
Dog	Scapula	120	68	52
	Humerus	178	90	88
	Femur	172	90	82
TOTAL		**1554**	**752**	**802**

**Table 2:** Dataset distribution and number of photos.

Animal Species	Number of Bones	Bone Type	Number of Images	Training	Testing	Validation
Cattle	62	Scapula	2744	2332	274	137
Cattle	58	Humerus	2744	2332	274	137
Cattle	60	Femur	2744	2332	274	137
Horse	52	Scapula	2744	2332	274	137
Horse	56	Humerus	2744	2332	274	137
Horse	52	Femur	2744	2332	274	137
Dog	42	Scapula	2744	2332	274	137
Dog	38	Humerus	2744	2332	274	137
Dog	40	Femur	2744	2332	274	137
TOTAL	460	9 Classes	24,696	20,988	2466	1233

**Table 3:** Configuration and hyperparameters of YOLO.

YOLO Hyperparameters	Value	Description
Learning Rate (lr0)	0.01	Initial learning rate
Final LR Factor (lrf)	0.1	Final learning rate multiplier
Momentum	0.937	SGD momentum
Weight Decay	0.0005	L2 regularization
Warmup Epochs	3.0	Number of warmup epochs
Box Loss Weight	0.05	Bounding box loss weight
Class Loss Weight	0.3	Classification loss weight
Object Loss Weight	0.7	Objectless loss weight
IoU Threshold	0.2	IoU threshold value
Mosaic Augmentation	1.0	Mosaic augmentation ratio
Mix-up	0.15	Mix-up augmentation ratio
Horizontal Flip	0.5	Horizontal flip probability
HSV-H	0.015	Hue variation
HSV-S	0.7	Saturation variation
HSV-V	0.4	Value/brightness variation

**Table 4:** Training configuration and hyperparameters.

Parameter	ResNet34	SmallCNN	AlexNet	Rationale
Learning Rate	0.001	0.001	0.001	Optimal convergence rate
Batch Size	32	32	32	Memory efficiency balance
Epochs	100	100	100	Sufficient convergence time
Optimizer	Adam	Adam	Adam	Adaptive learning rates
β_1_ADAM	0.9	0.9	0.9	Adaptive learning rates
β_2_ADAM	0.999	0.999	0.999	Standard RMSprop term
Weight Decay	1 × 10^−4^	1 × 10^−4^	1 × 10^−4^	L2 regularization
Loss Function	CrossEntropy	CrossEntropy	CrossEntropy	Multi-class classification
LR Scheduler	StepLR	StepLR	StepLR	Learning rate decay
Step Size	30	30	30	Scheduler step interval
Gamma	0.1	0.1	0.1	Learning rate decay factor
Early Stopping	Yes (patience15)	Yes (patience15)	Yes (patience15)	Overfitting prevention
Class Weight	Balanced	Balanced	Balanced	Class imbalance handling

**Table 5:** Performance evaluation of the YOLOv5s model for bone detection across different datasets.

Dataset	Images	Precision (%)	Recall (%)	mAP@0.5 (%)	F1-Score (%)
Training	1321	99.9	97.2	97.1	98.5
Testing	155	99.8	96.9	96.6	98.3
Validation	78	99.7	96.5	96.4	98.1

**Table 6:** Comparative performance metrics of the models used.

Model	Accuracy (%)	F1-Score (%)	Precision (%)	Recall (%)
ResNet34	97.6	97.2	96.8	97.4
SmallCNN	95.0	94.6	94.3	94.8
AlexNet	91.3	90.2	89.9	90.4

**Table 7:** Satisfaction scores by year of students (Q: question) (*p* > 0.05).

Year of Student	n	Q1	Q2	Q3	Q4	Q5	Mean
1st Year	45	4.87 ± 0.43	4.78 ± 0.59	4.91 ± 0.37	4.73 ± 0.58	4.93 ± 0.35	**4.84 ± 0.35**
2nd Year	30	4.83 ± 0.48	4.77 ± 0.57	4.90 ± 0.40	4.67 ± 0.61	4.90 ± 0.40	**4.81 ± 0.38**
3rd Year	25	4.76 ± 0.60	4.72 ± 0.68	4.88 ± 0.44	4.60 ± 0.71	4.88 ± 0.44	**4.77 ± 0.44**
4th Year	25	4.72 ± 0.61	4.64 ± 0.76	4.80 ± 0.50	4.52 ± 0.77	4.84 ± 0.47	**4.70 ± 0.48**
5th Year	25	4.80 ± 0.50	4.68 ± 0.69	4.84 ± 0.47	4.56 ± 0.71	4.88 ± 0.44	**4.75 ± 0.42**

**Table 8:** Detailed results of the student satisfaction survey (n = 150) (*p* > 0.05).

Survey Question (n = 150)	Mean ± SS	1 (%)	2 (%)	3 (%)	4 (%)	5 (%)	Positive Response (4 + 5) (%)
Q1. The application was easy to use	4.81 ± 0.52	0.7	0.0	1.3	14.0	84.0	98.0
Q2. Voice and text-based queries functioned accurately enough	4.73 ± 0.63	1.3	0.7	4.0	18.0	76.0	94.0
Q3. The application’s anatomical information was satisfactory	4.87 ± 0.43	0.0	0.7	2.0	8.0	89.3	97.3
Q4. The PDF export feature was useful	4.64 ± 0.67	1.3	2.0	6.0	20.7	70.0	90.7
Q5. I was satisfied with the overall performance of the application	4.87 ± 0.41	0.0	0.7	2.0	8.0	89.3	97.3

## Data Availability

The data that support the findings of this study are available from the corresponding author upon reasonable request.
